# Molecular analysis of HBV genotypes and subgenotypes in the Central-East region of Tunisia

**DOI:** 10.1186/1743-422X-7-302

**Published:** 2010-11-04

**Authors:** Naila Hannachi, Nadia Ben Fredj, Olfa Bahri, Vincent Thibault, Asma Ferjani, Jawhar Gharbi, Henda Triki, Jalel Boukadida

**Affiliations:** 1 Laboratoire de Microbiologie-Immunologie, UR02SP13, Hôpital Farhat Hached, Sousse, Tunisia; 2 Laboratoire de Virologie Clinique, Institut Pasteur Tunis, Tunis-Belvedere, Tunisia; 3 Laboratoire de Virologie, Groupe Hospitalier Pitié-Salpêtrière, Paris, France; 4 Laboratoire de Séquençage, Institut Supérieur de Biotechnologie de Monastir. Monastir, Tunisia

## Abstract

**Background:**

In Tunisia, country of intermediate endemicity for Hepatitis B virus (HBV) infection, most molecular studies on the virus have been carried out in the North of the country and little is known about other regions. The aim of this study was to determine HBV genotype and subgenotypes in Central-East Tunisia. A total of 217 HBs antigen positive patients were enrolled and determination of genotype was investigated in 130 patients with detectable HBV DNA. HBV genotyping methods were: PCR-RFLP on the pre-S region, a PCR using type-specific primers in the S region (TSP-PCR) and partial sequencing in the pre-S region.

**Results:**

Three genotypes (D, B and A) were detected by the PCR-RFLP method and two (D and A) with the TSP-PCR method, the concordance between the two methods was 93%. Sequencing and phylogenetic analysis of 32 strains, retrieved the same genotype (D and A) for samples with concordant results and genotype D for samples with discordant results. The sequences of discordant genotypes had a restriction site in the pre-S gene which led to erroneous result by the PCR-RFLP method. Thus, prevalence of genotype D and A was 96% and 4%, respectively. Phylogenetic analysis showed the predominance of two subgenotypes D1 (55%) and D7 (41%). Only one strain clustered with D3 subgenotype (3%).

**Conclusions:**

Predominance of subgenotype D7 appears to occur in northern regions of Africa with transition to subgenotype D1 in the East of the continent. HBV genetic variability may lead to wrong results in rapid genotyping methods and sequence analysis is needed to clarify atypical results.

## Background

Hepatitis B virus (HBV) infection is one of the major global health problems; more than 400 million persons are chronically infected by HBV with high risk of cirrhosis and hepatocellular carcinoma (HCC) [1]. Several viral factors influence the outcome of the infection such as DNA levels, viral mutations and HBV genotypes [2,3]. Based on sequence divergence in the entire genome, eight genotypes (A to H), differing by at least 8%, have been identified [4,5]. Genotypes A to D and F have been, recently, divided into multiple sub-genotypes with a difference ranging from 4 to 8% in their nucleotide sequences [1,3]. Sequencing is the gold standard to classify HBV genotypes and sub-genotypes; however, the method is expensive and fastidious [5]. To overcome this problem, different techniques have been developed, based on either PCR with type-specific primers, PCR with restriction fragment length polymorphism (RFLP) or PCR-hybridization probe [6-8,5]. These rapid molecular methods have been performed in many countries for epidemiological studies.

HBV genotypes have a characteristic geographical distribution: genotype A is prevalent in Europe, India, Africa and America. Genotypes B and C are predominant in China, Japan and Southern Asia whereas genotype D is widespread in the Mediterranean area and the Middle East region. Genotype E is found in patients from West Africa and genotype F in Central and South America. Genotype H has been described in Mexico and Central America. Genotype G has been first identified in France and the United States, and was recently detected in Mexico [2].

Tunisia is a country with an intermediate HBV endemicity; prevalence of HBsAg range from 4 to 7% in the general population [9]. The rate of HBsAg positivity varies widely from the north to the south of the country [9,10]. Previous studies reported predominance of genotype D (over than 80%) with limited circulation of genotypes A, B, C and E [11,12]. For HBV subgenotypes, only one study has been previously conducted with description of a novel subgenotype named D7 [13]. All these molecular studies were performed in the north part of the country; no data are yet available in the other regions.

The present work aimed to complete Tunisian data on HBV genotypes and subgenotypes circulation. For this purpose, this study was conducted on HBV infected patients originating from the central part of Tunisia. Two molecular approaches based either on a multiplex-PCR using specific primers or RFLP were used to identify HBV genotypes. Partial sequencing was performed to confirm the results obtained by these methods and to study HBV subgenotypes.

## Study design

### Studied population

Our population included 217 patients infected by HBV and recruited during the period from September 2007 to September 2008. All of these patients were previously tested for HBsAg by immuno-enzymatic test (Abbott AXSYM(r) HBsAg Assay) and were positive for this marker. Patients aged from 7 to 80 year-old (mean age 36.38 ± 14.26 years) with a M/F sex ratio of 0.68. They attended different primary care centers in the central region of Tunisia (governorates of Sousse, Monastir, Mahdia and Kairouan). Six patients with chronic hepatitis and two with cirrhosis were positive for HBeAg (Table 1).

**Table 1:** HBe Ag positivity and HBV DNA detection according to clinical status

		Positive HBe Ag		Positive HBV DNA	
Clinical status	N (%)	N	%	N	%
Acute hepatitis	2 (1%)	2	-	2	-
Inactive carriers	162 (74.6%)	0	0%	82	50.6%
Chronic hepatitis	40 (18.4%)	6	15%	39	97.5%
Cirrhosis	13 (6%)	2	15,38	7	53.8

### Viral DNA extraction and genotyping

HBV DNA was extracted from 200 μl of serum samples using QIAamp DNA blood kit (Qiagen, Chatsworth, CA). HBV DNA was detected by PCR amplification of the fragment located between nucleotides 2823 and 80 in the Pre-S region of HBV genome, as described by Lindh et al [7]. The sensitivity of this method was previously estimated to be 10^3 ^copies/ml.

Two genotyping methods were used:

- RFLP analysis of the fragment obtained by PCR amplification in the Pre-S region: the amplification product was digested separately by AvaII and DpnII restriction enzymes with separation of the resulting DNA fragments by electrophoresis in a 4% agarose gel stained by ethidium bromide. Genotypes A to F of HBV were identified by the obtained restriction patterns according to Lindh et al [7].

- PCR amplification using type-specific primers (TSP-PCR) described previously by Naito et al [6]: it is a nested PCR with a first amplification of a 1063 fragment located between nucleotides 2823 and 704 in the Pre-S and S regions of the genome followed by a second amplification with two separate mixtures A and B. These mixtures allow specific detection of genotypes A (68 bp), B (281 bp), and C (122 bp) for the first one and genotypes D (119 bp), E (167 bp), and F (97 bp) for the second.

These two genotyping methods are unable to identify genotype G and H. Both HBV genotyping methods were performed on all patients' specimens.

Sequencing was performed with a BigDye Terminator Cycle Sequencing kit on an ABI 3130 automated sequencer (Applied Biosystems, Darmstadt, Germany), with the same primers as those used for PCR amplification of pre-S region. The sequences obtained were compared with published sequences from the same genomic region available in GenBank.

Alignment was performed using CLUSTAL W method in MEGA 4.1 software. Phylogenetic trees were constructed using the neighbour-joining algorithm of MEGA4.1. software, with 1000 Bootstrap replicates.

## Results

Sixty percent of patients (n = 130 out of 217) were positive by PCR amplification in the pre-S region. HBV DNA was detected for all patients with positive HBeAg and for 58% with positive anti-HBe sera. Table 1 shows the HBe Ag status and the PCR amplification results in the studied population.

**Figure 1:**
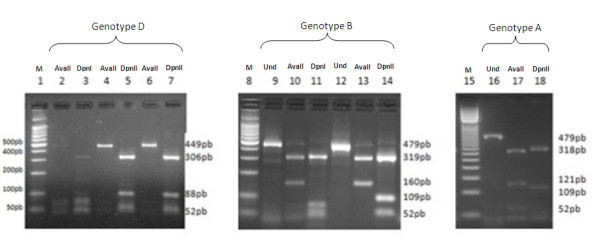
**Genotyping of HBV by the RFLP-PCR method**. *Ava II : pre-S region digested with AvaII; DpnII: pre-S region digested with DpnII;* *Und: undigested pre-S fragment; M: molecular size standards*,

PCR-RFLP and TSP-PCR were successfully assessed for the 130 samples with detectable HBV DNA. Three HBV genotypes were detected by PCR-RFLP: D, A and B (Figure 1). Genotype D was observed in 89% of the cases with a restriction pattern corresponding to D2 (undigested with Ava II and bands 306 pb, 88 pb, 52 pb with Dpn II). Genotype A was detected in 4% of the cases with specific RFLP profile of A1 pattern (301 pb, 121 pb, 57 pb with AvaII and 318 pb, 109 pb and 52 pb with DpnII). In 7% of the cases, a restriction profile corresponding to genotype B was observed (matching with B6 pattern: 319 pb and 160 bp with Ava II and 318 pb, 109 pb and 52 pb with DpnII). This profile was observed in 5 cirrhotic cases.

Different results were provided by TSP-PCR: only two genotypes, D and A, were detected in 96% and 4% of the samples, respectively. Concordance with PCR-RFLP was found in 93% of the cases (121/130). The nine cases classified as genotype B by PCR-RFLP were identified as genotype D by TSP-PCR.

**Figure 2:**
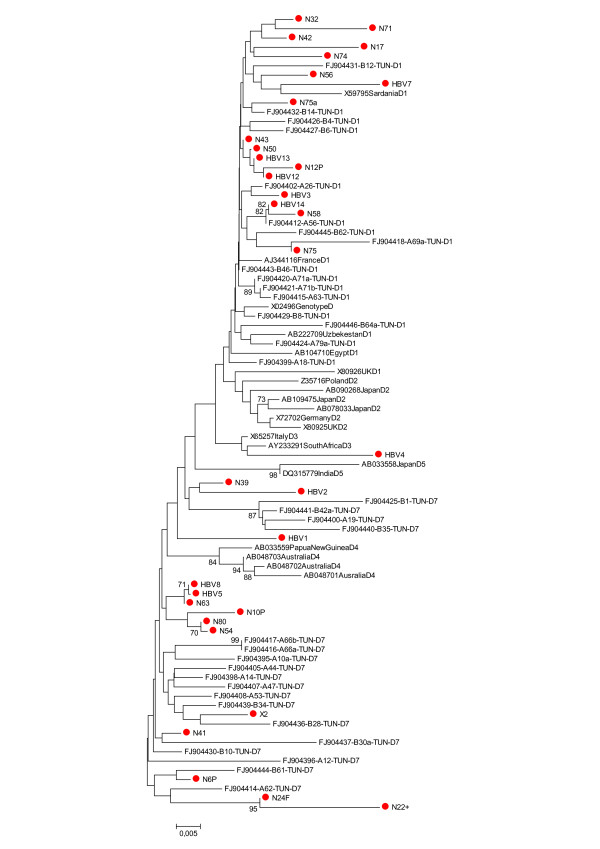
**Phylogenetic analysis based on 86 sequences of 431 nucleotides within the HBsAg region**. *The evolutionary history was inferred using the Neighbor-Joining method. The evolutionary distances were computed using the Maximum Composite Likelihood method and are in the units of the number of base substitutions per site. There were a total of 431 positions in the final dataset. Phylogenetic analyses were conducted in MEGA4. Sequences from patients included in this study are labeled with a red dot. Reference sequences are indicated by their accession number*.

Partial sequencing in the pre-S region was performed for 32 samples: seven of nine samples with discordant results by the two methods used and 25 with concordant results (24 with genotype D and 1 with genotype A). Figure 2 shows a phylogenetic tree obtained after comparison with selected sequences from GenBank. Phylogenetic analysis confirmed the concordant results between the two genotyping methods. For the 7 samples with discordant genotype results, the genotype determined through sequencing was D (Table 2). Thus, real frequencies of genotype D and A strains were 96% and 4% respectively.

**Table 2:** Clinical status, subgenotype and genotype of HBV strains according to the genotyping method.

		Genotype determined using			
			
Sample	Clinical status	PCR-RFLP	TSP-PCR	Sequencing	Subgenotype
1	Inactive carrier	D	D	D	D1
2	Inactive carrier	D	D	D	D1
3	Inactive carrier	D	D	D	D1
4	Inactive carrier	D	D	D	D1
5	Inactive carrier	D	D	D	D1
6	Chronic hepatitis	D	D	D	D1
7	Chronic hepatitis	D	D	D	D1
8	Chronic hepatitis	D	D	D	D1
9	Chronic hepatitis	D	D	D	D1
10	Chronic hepatitis	D	D	D	D1
11	Chronic hepatitis	D	D	D	D1
12	Chronic hepatitis	D	D	D	D1
13	Chronic hepatitis	D	D	D	D1
14	Chronic hepatitis	D	D	D	D1
15	Chronic hepatitis	D	D	D	D1
16	Inactive carrier	D	D	D	D7
17	Inactive carrier	D	D	D	D7
18	Inactive carrier	D	D	D	D7
19	Inactive carrier	D	D	D	D7
20	Chronic hepatitis	D	D	D	D7
21	Chronic hepatitis	D	D	D	D7
22	Chronic hepatitis	D	D	D	D7
23	Chronic hepatitis	D	D	D	D7
24	Cirrhosis	D	D	D	D7
25	Cirrhosis	**B**	D	D	D1
26	Cirrhosis	**B**	D	D	D1
27	Chronic hepatitis	**B**	D	D	D7
28	cirrhosis	**B**	D	D	D7
29	cirrhosis	**B**	D	D	D7
30	cirrhosis	**B**	D	D	D7
31	Chronic hepatitis	**B**	D	D	D 3
32	Inactive carrier	A	A	A	-

Discordant results are shaded.

Analysis of the region located between nucleotides 2823 and 80 in pre-S gene in samples giving erroneous result with the RFLP method revealed the presence of an additional restriction site for AvaII in our sequences. The new restriction site resulted in an additional fragment of 160 pb. The presence of the same site of restriction in this region of genotype D strains was also observed in 8 sequences available in GenBank under the following assession numbers: DQ464170, EU594406, AB109478, AY796031, FJ349235, FJ001987, FJ904365 and FJ904433[13-17].

Among the 31 genotype D strains, phylogenetic analysis showed predominance of subgenotypes D1 (54%). A total of 13 samples (41%) clustered into the novel D7 subgenotype group and just one belonged to D3 subgenotype (3%).

## Discussion

Our study, conducted for the first time in a population from the Central-East of Tunisia, identified genotype D as the most prevalent in this region. These findings are concordant with previous studies conducted in the North of the country where genotype D was detected in more than 90% of chronic hepatitis B cases [11-13]. All these results from different regions confirm that genotype D is largely circulating in the country. Globally, this genotype is known by its high prevalence in the Mediterranean area, the near and middle east, and south Asia and its high risk of provoking fulminant hepatitis. It is also responsible of severe chronic liver diseases more frequently than other genotypes [1,3,18]. Genotype D is also known to be frequently associated with pre-core mutants which increase the risk of evolution to cirrhosis and HCC [11]. This type of mutants seems to be frequent in our population in view of the fact that 59% of the patients are characterized by the absence of HBeAg but detectable HBV DNA.

Beside genotype D, only genotype A was detected from a few samples in our study. Genotype A was previously identified in 6 to 8% of Tunisian patients in co-circulation with genotypes B, C and E [11-13]. The lack of detection of these latest genotypes, in our study, could be explained by the origin of our patients which was different from what described previously or by the techniques used which have different sensitivity to detect genotypes [19]. Indeed, genotype G and H were not investigated for all patients because the two rapid methods do not allow identification of these genotypes. Yet, sequencing performed for 32 of our strains did not objective their presence. As reported by previous studies conducted in Tunisians, these two genotypes seem to be not circulating in our country. However, more investigations should be performed, especially for genotype G which was previously described in a Mediterranean country (France) [2].

For HBV genotyping, three methods were used, PCR-RFLP, TSP-PCR and partial sequencing; discordant results were observed between PCR-RFLP and TSP-PCR especially for genotype B. Discordance between these two methods can be related to the high variability of HBV genome which results in changes of enzyme restriction sites, suppressing known sites or creating new ones. These later modifications may lead to erroneous results with RFLP methods [19,20]. Analysis of our sequences is in agreement with this methodological artefact. Indeed, it revealed the presence of an additional restriction site for Ava-II in the pre-S region which has not been described previously [7]. Furthermore, this additional site is also present in other sequences deposited in Genbank by several authors [13-17]. This restriction site resulted, in our study, in an additional fragment of 160 pb with a RFLP pattern of genotype B but it did not interfere with results of other studies relying on sequencing methods and which succeeded in identifying genotype D [13-17]. For this reason, results obtained by PCR-RFLP should be carefully analyzed because introduction of new restriction site in targeted sequences can lead to erroneous results with this technique [21]. The genotyping based on this RFLP method is largely used for epidemiological studies because it is easy to perform but this approach suffers some limits especially in area with high prevalence of genotype D [22-25].

Sequencing, performed for discordant samples, gave fully concordant results to those obtained by TSP-PCR; thus, it reveals the high efficiency of this later method [6,7]. Lim et al have also previously reported more specific results by TSP-PCR in comparison to a PCR-RFLP based method described by Lindh et al. in 1998 [26]. The principal advantage of TSP-PCR is the region of the genome targeted by this method; in fact, TSP-PCR amplifies part of the S region which is known to be more accurate for genotypic determination than the pre-S gene amplified by PCR-RFLP [26,27]. The bias observed between the two PCR based methods could then be simply due to the region used for genotyping.

In addition to the risk of erroneous results, the limit of the rapid methods using classic PCR is the low sensitivity of DNA detection (the limit of detection of our method being 10^3 ^copies/ml). This explains that the genotyping method was performed for only 60% of positive HBs Ag patients in our study. Due to this lower sensitivity, one cannot exclude that genotype distribution studied with our method may be slightly biased on samples with higher viral load. Confirmation of our findings with a more sensitive technique would be of interest.

Phylogenetic analysis and comparison to other Tunisian sequences of genotype D revealed high identity between sequences and identified two subgenotypes for our patients, D7 (41%) and D1 (56%). D7 is a novel subgenotype identified, for the first time, by Meldal et al in 59% of Tunisian patients [13]. Data from Algeria and Morrocco suggested the predominance of this new subgenotype in the region [23,28]. Subgenotype D1 is predominant in the Eastern part of Africa; Saudy et al described it in Egypt [29]. Our region geographically located between northern Africa and the east of the continent seems to be a transition zone between subgenotype D7 and subgenotype D1. Subgenotype D3 was observed in only few cases in our study and is related to Italian sequences; this result reflects probably regular human migration between Tunisia and Italy. Limitation of our study is obviously the relative short fragment studied to construct our phylogenetic analyses with the risk of poor discrimination between subtypes or even misclassification. Although this approach might be sufficient for screening, complete genome based genotyping is certainly required for accurate classification. In our study we did not find any correlation between clinical status and D subgenotype; further works including a larger proportion of inactive carriers are needed to confirm our findings.

In conclusion, our study completes previous Tunisian data and confirms the predominance of genotype D and subgenotypes D1 and D7. Our comparison between two simple genotyping methods that are largely used for epidemiological studies demonstrates the importance of sequencing to confirm results when the results are discordant.

## Abbreviations

**HBV: **Hepatitis B Virus; **DNA: **Desoxyribonucleic Acid; **HBs Ag: **Hepatitis B surface Antigen; **HBe Ag: **HBe Antigen; **anti-HBe: **anti-HBe antibodies; **PCR: **Polymerase Chain Reaction; **bp: **base pair; **RFLP: **restriction fragment length polymorphism; **TSP-PCR: **PCR amplification using type-specific primers.

## Competing interests

The authors declare that they have no competing interests.

## Authors' contributions

NH and OB conceived of the study, participated in its design, and in drafting the manuscript. HT and JB participated in the study design and coordination and in discussing manuscript. NH, NBF and OB carried out the serological tests, molecular genetic studies; participated in data analysis and in the sequence alignment and drafted the manuscript. VT participated in the sequence alignment, in phylogenetic analysis and drafted the manuscript. JG and AF participated in the sequence analysis. All authors read and approved the final manuscript.

## References

[B1] Günther S Genetic variation in HBV infection: genotypes and mutants J Clin Virol 2006 36 S3 S11 10.1016/S1386-6532(06)80002-8 16831690

[B2] Schaefer S Hepatitis B virus taxonomy and hepatitis B virus genotypes World J Gastroenterol 2007 13 1 14 21 17206751 10.3748/wjg.v13.i1.14 PMC4065870

[B3] McMahon BJ The influence of hepatitis B virus genotype and subgenotype on the natural history of chronic hepatitis B Hepatol Int 2009 3 2 334 42 10.1007/s12072-008-9112-z 19669359 PMC2716762

[B4] Kidd-Ljunggren K Miyakawa Y Kidd AH Genetic variability in hepatitis B viruses J Gen Virol 2002 83 1267 80 12029141 10.1099/0022-1317-83-6-1267

[B5] Miyakawa Y Mizokami M Classifying hepatitis B virus genotypes Intervirology 2003 46 6 329 38 10.1159/000074988 14688448

[B6] Naito H Hayashi S Abe K Rapid and specific genotyping system for hepatitis B virus corresponding to six major genotypes by PCR using type-specific primers J Clin Microbiol 2001 39 362 364 10.1128/JCM.39.1.362-364.2001 11136801 PMC87732

[B7] Lindh M Gonzalez JE Norkrans G Horal P Genotyping of hepatitis B virus by restriction pattern analysis of a pre-S amplicon J Virol Methods 1998 72 2 163 74 10.1016/S0166-0934(98)00026-3 9694324

[B8] Kato H Ruzibakiev R Yuldasheva N Hegay T Kurbanov F Achundjanov B Tuichiev L Usuda S Ueda R Mizokami M Hepatitis B virus genotypes in Uzbekistan and validity of two different systems for genotyping J Med Virol 2002 67 477 483 10.1002/jmv.10126 12115992

[B9] Triki H Said N Ben Salah A Arrouji A Ben Ahmed F Bouguerra A Hmida S Dhahri R Dellagi K Seroepidemiology of hepatitis B, C and delta viruses in Tunisia Trans R Soc Trop Med Hyg 1997 91 1 11 14 10.1016/S0035-9203(97)90374-6 9093616

[B10] Ben-Alaya-Bouafif N Bahri O Chlif S Bettaieb J Toumi A Bel Haj HN Zâatour A Gharbi A Dellagi K Triki H Ben Salah A Heterogeneity of hepatitis B transmission in Tunisia: risk factors for infection and chronic carriage before the introduction of a universal vaccine program Vaccine 2010 28 19 3301 7 10.1016/j.vaccine.2010.02.101 20226251

[B11] Bahri O Cheikh I Hajji N Djebbi A Maamouri N Sadraoui A Mami NB Triki H Hepatitis B genotypes, precore and core promoter mutants circulating in Tunisia J Med Virol 2006 78 3 353 357 10.1002/jmv.20554 16419125

[B12] Ayed K Gorgi Y Ayed-Jendoubi S Aouadi H Sfar I Najjar T Ben Abdallah T Hepatitis B virus genotypes and precore/core-promoter mutations in Tunisian patients with chronic hepatitis B virus infection J Infect 2007 54 3 291 297 10.1016/j.jinf.2006.05.013 16911832

[B13] Meldal BH Moula NM Barnes IH Boukef K Allain JP A novel hepatitis B virus subgenotype, D7, in Tunisian blood donors J Gen Virol 2009 90 Pt 7 1622 1628 10.1099/vir.0.009738-0 19339480

[B14] Tallo T Norder H Tefanova V Krispin T Priimägi L Mukomolov S Mikhailov M Magnius LO Hepatitis B virus genotype D strains from Estonia share sequence similarity with strains from Siberia and may specify ayw4 J Med Virol 2004 74 2 221 7 10.1002/jmv.20169 15332270

[B15] Michitaka K Tanaka Y Horiike N Duong TN Chen Y Matsuura K Hiasa Y Mizokami M Onji M Tracing the history of hepatitis B virus genotype D in western Japan J Med Virol 2006 78 1 44 52 10.1002/jmv.20502 16299716

[B16] Bozdayi G Türkyilmaz AR Idilman R Karatayli E Rota S Yurdaydin C Bozdayi AM Complete genome sequence and phylogenetic analysis of hepatitis B virus isolated from Turkish patients with chronic HBV infection J Med Virol 2005 76 4 476 81 10.1002/jmv.20386 15977237

[B17] Pourkarim MR Amini-Bavil-Olyaee S Verbeeck J Lemey P Zeller M Rahman M Maes P Nevens F Van Ranst M Molecular evolutionary analysis and mutational pattern of full-length genomes of hepatitis B virus isolated from Belgian patients with different clinical manifestations J Med Virol 2010 82 3 379 89 10.1002/jmv.21726 20087936

[B18] Cao GW Clinical relevance and public health signifcance of hepatitis B virus genomic variations World J Gastroenterol 2009 15 46 5761 5769 10.3748/wjg.15.5761 19998495 PMC2791267

[B19] Bartholomeusz A Schaefer S Hepatitis B virus genotypes: comparison of genotyping methods Rev Med Virol 2004 14 1 3 16 10.1002/rmv.400 14716688

[B20] Serin MS Akkiz H Abayli B Oksuz M Aslan G Emekdas G Genotyping of hepatitis B virus isolated from chronic hepatitis B patients in the south of Turkey by DNA cycle-sequencing method Diagn Microbiol Infect Dis 2005 53 1 57 60 10.1016/j.diagmicrobio.2005.04.007 16054327

[B21] Rodríguez-Nóvoa S Gómez-Tato A Aguilera-Guirao A Castroagudín J González-Quintela A Garcia-Riestra C Regueiro BJ Hepatitis B virus genotyping based on cluster analysis of the region involved in lamivudine resistance J Virol Methods 2004 115 1 9 17 10.1016/j.jviromet.2003.08.011 14656456

[B22] Eroglu C Leblebicioglu H Gunaydin M Turan D Sunbul M Esen S Sanic A Distinguishing hepatitis B virus (HBV) genotype D from non-D by a simple PCR J Virol Methods 2004 119 2 183 187 10.1016/j.jviromet.2004.03.003 15158601

[B23] Ezzikouri S Chemin I Chafik A Wakrim L Nourlil J Malki AE Marchio A Dejean A Hassar M Trepo C Pineau P Benjelloun S Genotype determination in Moroccan hepatitis B chronic carriers Infect Genet Evol 2008 8 3 306 12 10.1016/j.meegid.2008.01.010 18372221

[B24] Utama A Octavia TI Dhenni R Miskad UA Yusuf I Tai S Hepatitis B virus genotypes/subgenotypes in voluntary blood donors in Makassar, South Sulawesi, Indonesia Virol J 2009 19 6 128 10.1186/1743-422X-6-128 PMC2732614 19691824

[B25] Zekri AR Hafez MM Mohamed NI Hassan ZK El-Sayed MH Khaled MM Mansour T Hepatitis B virus (HBV) genotypes in Egyptian pediatric cancer patients with acute and chronic active HBV infection Virol J 2007 15 4 74 10.1186/1743-422X-4-74 PMC1947959 17631684

[B26] Lim CK Tan JT Ravichandran A Chan YC Ton SH Comparison of PCR-based genotyping methods for hepatitis B virus Malays J Pathol 2007 29 2 79 90 19108399

[B27] Mizokami M Nakano T Orito E Tanaka Y Sakugawa H Mukaide M Robertson BH Hepatitis B virus genotype assignment using restriction fragment length polymorphism patterns FEBS Lett 1999 450 1-2 66 71 10.1016/S0014-5793(99)00471-8 10350059

[B28] Khelifa F Thibault V Characteristics of hepatitis B viral strains in chronic carrier patients from North-East Algeria Pathol Biol (Paris) 2009 57 1 107 13 18835106 10.1016/j.patbio.2008.07.031

[B29] Saudy N Sugauchi F Tanaka Y Suzuki S Aal AA Zaid MA Agha S Mizokami M Genotypes and phylogenetic characterization of hepatitis B and delta viruses in Egypt 2003 70 4 529 36 12794714 10.1002/jmv.10427

